# Integrating Deep Learning Derived Morphological Traits and Molecular Data for Total-Evidence Phylogenetics: Lessons from Digitized Collections

**DOI:** 10.1093/sysbio/syae072

**Published:** 2025-01-18

**Authors:** Roberta Hunt, José L Reyes-Hernández, Josh Jenkins Shaw, Alexey Solodovnikov, Kim Steenstrup Pedersen

**Affiliations:** Department of Computer Science, University of Copenhagen, Universitetsparken 1, Copenhagen, 2100, Denmark; Natural History Museum of Denmark, Copenhagen DK-2100, Denmark; Natural History Museum of Denmark, Copenhagen DK-2100, Denmark; Natural History Museum of Denmark, Copenhagen DK-2100, Denmark; Department of Computer Science, University of Copenhagen, Universitetsparken 1, Copenhagen, 2100, Denmark; Natural History Museum of Denmark, Copenhagen DK-2100, Denmark

**Keywords:** Continuous morphological traits, cladistics, deep metric learning, image analysis, neural networks, phylogenetics, total evidence analysis

## Abstract

Deep learning has previously shown success in automatically generating morphological traits that carry a phylogenetic signal. In this paper, we explore combining molecular data with deep learning derived morphological traits from images of pinned insects to generate total-evidence phylogenies and we reveal challenges. Deep learning derived morphological traits, while informative, underperformed when used in isolation compared to molecular analyses. However, they can improve molecular results in total evidence settings. We use a dataset of rove beetle images to compare the effect of different dataset splits and deep metric loss functions on morphological and total evidence results. We find a slight preference for the cladistic dataset split and contrastive loss function. Additionally, we explore the effect of varying the number of genes used in inference and find that different gene combinations provide the best results when used on their own vs in total evidence analysis. Despite the promising nature of integrating deep learning techniques with molecular data, challenges remain regarding the strength of the phylogenetic signal and the resource demands of data acquisition. We suggest that future work focus on improved trait extraction and the development of disentangled networks to better interpret the derived traits, thus expanding the applicability of these methods in phylogenetic studies.

Phylogenetics or reconstructing the Tree of Life in detail is at the core of modern biology ([Bibr CIT0074][Bibr CIT0074]; Cavender-Bares et al.^2009^; [Bibr CIT0087][Bibr CIT0087]; Hassler et al.^2023^). Although in recent decades phylogenetic inference has been a high-throughput endeavor due to the availability of molecular data ([Bibr CIT0087][Bibr CIT0087]), for more than two centuries it was a practice based on the visual study of the anatomical details of the organisms ([Bibr CIT0022][Bibr CIT0022]). Despite the recent emphasis on molecular data in the phylogenetic analysis, morphology remains crucial ([Bibr CIT0022][Bibr CIT0022]; [Bibr CIT0048][Bibr CIT0048]; de Almeida et al.^2023^), particularly for including fossils and dating evolutionary events through a total evidence approach (Ronquist et al.^2012^; Zhang et al.^2015^; [Bibr CIT0065][Bibr CIT0065]). Contrary to molecular data, the high-throughput use of the morphological traits in statistical phylogenetics is strongly hindered by the complex nature of morphology and as a consequence lesser developed tools for its analysis ([Bibr CIT0076][Bibr CIT0076]). It is therefore noteworthy that recent research has shown that morphological traits automatically extracted from habitus images of the organisms using deep learning-based image analysis carry a phylogenetic signal (Cuthill et al.^2019^; [Bibr CIT0041][Bibr CIT0041]; [Bibr CIT0032][Bibr CIT0032]; Furusawa et al.^2023^). However, because the deep learning derived traits are not sliced into homologous and non-homologous traits (characters), this signal is not amplified as such and could be blurred with non-phylogenetic clustering.

Crafting a morphological data matrix for phylogenetic analysis manually, based on expert knowledge, is a tedious and time-consuming process. This task involves formulating traits (characters) and their respective states. It is achieved through comparisons of multiple species with diverse morphologies. Using extensive anatomical and taxonomic knowledge, an expert aligns only homologous character states for analysis across species. Cuthill et al. (2019) demonstrated that euclidean phenotypic distances calculated using a deep convolutional triplet network, captured the wing phylogeny pattern reflecting Müllerian mimicry and thus convergence between the interspecies co-mimics in the *Heliconius erato* and *H. melpomene* butterfly complex. However, would this method be capable of capturing other, less obvious patterns that reflect synapomorphic similarity, and therefore, an overall species placement within a phylogeny? Lacking the critical filter in the automatic capture of the phenotypic similarity in butterflies in this example and other organisms is an unsolved problem. Kiel (2021) well recognized the issue of convergent evolution that is “invisible” for the CNN he trained for assessing bivalve phylogeny from thousands of images of 75 bivalve families. Therefore, he trained two further CNNs on the same images but grouped by the orders and subclasses these families belonged to. He demonstrated that this improved the inferred phylogenetic relationships even for families, which these extra CNNs were not trained for. Furusawa et al. (2023) presented the morphologically regulated variational AutoEncoder (Morpho-VAE) for analyzing shapes of primate mandibles to overcome the difficulty of encoding shapes usually tediously done by comparing anatomically prominent landmarks. Their goals were not phylogenetic but they showed that their automatically extracted morphological features reflected the families to which the organisms belong, i.e., they were phylogenetically meaningful. These experiments show that further exploration of how to overcome the convergency problem in the automatic capture of phenotypic data for phylogenetic analysis is promising and needed.

In expert-based data capture, beyond the substantial time and expertise required, the final phylogenetic framework itself informs us about which similarities are homology-based (synapomorphic) and which are homoplastic. Developing rigorous and automated methods for data capture is essential for reducing subjectivity and enhancing efficiency in phylogenetic analysis. If improved, these methods could integrate seamlessly with mass digitization initiatives in major natural history collections, which are generating millions of specimen images ([Bibr CIT0004][Bibr CIT0004]; [Bibr CIT0072][Bibr CIT0072]; Wilson et al.^2023^). Such integration could revolutionize phylogenetics by making research scalable and widely accessible. Therefore, it is of great interest to determine how this signal could be strengthened and how it might be integrated into existing methodologies. In this paper, we explore these methods using a dataset of habitus images (interpreted here as the dorsal view of the entire beetle body) of rove beetles from museum collections. Our goal is to assess the strength of the phylogenetic signal captured and to determine how deep-learning-derived morphological traits from digitized specimen images could contribute to phylogenetic inference, either independently or in combination with molecular data. Before presenting our methods and results, we first introduce several key themes and considerations that guided our study design.

## Is Habitus Enough?

Since Linnaeus (Linné et al.^1788^), if not before, hierarchical grouping has been at the core of understanding the diversity of life on Earth with the evolutionary theory elevating the importance of constructing genealogical relationships to decipher the connections among both extant and extinct species. The "Tree of life" became a universal metaphor depicting relationships between living and extinct organisms ([Bibr CIT0055][Bibr CIT0055]; [Bibr CIT0030][Bibr CIT0030]). Historically, in the absence of sophisticated optical and anatomical instruments, the habitus served as the primary means for comparing organismal phenotypes. However, evolutionary biology’s advancements have highlighted the complexity of phenotypes, underscoring that the habitus represents merely a subset of an organism’s morphological information. The maturing concepts of homology guided the division of a holistic phenotype into single morphological traits and their assessment as shared ancestry or, on the contrary, cases of convergence, a division crucial in phylogenetics ([Bibr CIT0080][Bibr CIT0080]). In modern statistical phylogenetics, intuitive or even statistical phenetic assessment of the overall morphological similarity among organisms ([Bibr CIT0073][Bibr CIT0073]) gave way to the analysis of morphology partitioned into homological characters ([Bibr CIT0024][Bibr CIT0024]). A concept of a morphological trait and its homological states in phylogenetics is rather complex ([Bibr CIT0082][Bibr CIT0082]) and raises questions about data automatically obtained from the specimen images. The suitability of utilizing images capturing the external morphology, or habitus, of organisms for phylogenetic analysis warrants initial examination.

## The Role of Continuous Traits in Phylogenetics

The first stochastic process model of the evolution of continuous trait data on a phylogeny was Brownian motion, proposed early in the history of statistical phylogenetics ([Bibr CIT0016][Bibr CIT0016]; [Bibr CIT0017][Bibr CIT0017]). Among the great diversity of morphological characters used in phylogenetics, continuous characters of shape are rare due to lack of tools for their proper coding and assessment. The majority of homological morphological traits used in the phylogenetic analysis are the so-called discrete characters ([Bibr CIT0052][Bibr CIT0052]). However, studies suggest that continuous characters are informative and may even be preferable over discrete characters ([Bibr CIT0062][Bibr CIT0062]).

## The Growing Role of Computer Vision in Phylogenetics

Advancements in computer vision and machine learning are enabling the collection of morphological data for phylogenetic analysis at a scale similar to phylogenomics. With digital images, algorithms can quickly convert these into numerical vectors that mimic DNA sequences, providing a new form of data for analysis. Numerical vectors automatically generated from habitus photos return us to an early trend of the morphology-based phylogenetics called phenetics, which was abandoned early in the history of statistical phylogenetics for several important reasons ([Bibr CIT0036][Bibr CIT0036]). However, phenetics played an important role as an early stepping stone towards modern phylogenetics and perhaps it was abandoned due to the lack of good tools to generate informative data at the time. Also, the idea that the overall similarity between taxa may have a phylogenetic signal if properly assessed was never rejected and does have support, especially when dealing with lower taxonomic categories ([Bibr CIT0036][Bibr CIT0036]).

## The Role of Deep Learning

Deep learning, a subfield of machine learning, has proven to be an effective method to extract traits across various fields, from natural language processing (Otter et al.^2021^) to image processing ([Bibr CIT0037][Bibr CIT0037]). In the field of entomology, deep learning has proven quite successful at classification and quantification of mimicry (Valan et al.^2019^; Kelly et al.^2021^; Høye et al.^2021^; MacLeod et al.^2022^; [Bibr CIT0050][Bibr CIT0050]; [Bibr CIT0063][Bibr CIT0063]), and has already been applied to extracting morphological traits from images of various animals for phylogenetics (Cuthill et al.^2019^; [Bibr CIT0041][Bibr CIT0041]; [Bibr CIT0032][Bibr CIT0032]; Furusawa et al.^2023^). As the field of deep learning has grown over the last decade, there are thousands of model architectures, loss functions, and parameters to choose from, and testing them all is not feasible. Instead, this paper focuses on how simpler methods of boosting the phylogenetic signal might be completed. Therefore, for simplicity, we use only one well-known architecture, ResNet50 (He et al.^2016^), and a few selected loss functions commonly used in the field of deep metric learning (Roth et al.^2020^).

Deep metric learning, an advanced subfield of machine learning, focuses on understanding and quantifying the similarity or dissimilarity between data points in a high-dimensional space. By leveraging deep neural networks, this approach aims to learn a distance function that effectively maps data points such that similar items are pulled closer together while dissimilar items are pushed apart in the latent space. This methodology is particularly pertinent to phylogenetic trait extraction over traditional classification models due to its ability to capture nuanced relationships and continuous variations among biological species. Unlike classification models, which categorize data into discrete classes and often overlook the intricate relationships between them, deep metric learning accommodates the complexity and continuity of evolutionary traits. This makes it exceptionally suitable for phylogenetics, where the objective is to unravel the evolutionary distances and ancestral linkages among species. By focusing on relative distances rather than absolute categorizations, deep metric learning facilitates a more nuanced understanding of phylogenetic traits, enabling researchers to uncover subtle evolutionary patterns that classification models might miss. Loss functions utilized in deep metric learning can be categorized into three distinct types: ranking-based, classification-based, and proxy-based approaches. Ranking-based approaches modulate the latent space by endeavoring to minimize the distance between analogous images and maximize the separation between dissimilar images within this space. Classification-based approaches presuppose that executing a classification task will inherently organize the latent space into clusters. Conversely, proxy-based approaches create a distribution for each class and evaluate each data point relative to these distributions. To ensure a comprehensive understanding, our investigation encompasses loss functions from each of these categories. It is important to acknowledge that deep learning methodologies have been extensively applied to the inference of phylogenetic relationships using molecular traits. A detailed survey of these applications is provided in Mo et al. (2024).

## Methods of Phylogenetic Inference

Studies such as the present one would ideally compare different statistical methods or approaches to phylogenetic reconstruction. Each phylogenetic method has different strengths and weaknesses and is highly dependent on the nature of the data, philosophical viewpoint of the practitioner and available computational resources ([Bibr CIT0087][Bibr CIT0087]). Despite Bayesian Inference being a widely used method for inferring phylogenetic relationships based on molecular (Huelsenbeck et al.^2001^) and morphological ([Bibr CIT0084][Bibr CIT0084]) data, we experienced some constraints in analyzing continuous trait data within a Bayesian framework. As far as we know, of all the Bayesian phylogenetic Inference software only RevBayes (Höhna et al.^2016^) currently supports using continuous trait data, however it does not yet support missing values/taxa in said data ([Bibr CIT0088][Bibr CIT0088]). Therefore due to ease we present our results using only Maximum Parsimony ([Bibr CIT0019][Bibr CIT0019]).

## Our Contributions

In this paper, we show how deep learning can be used to extract continuous morphological traits that carry a phylogenetic signal, and how these traits can be used independently or combined with molecular data in a total-evidence framework. We compare and evaluate the results of these analyses across different methodological choices.

### Materials and Methods

**Fig 1:**
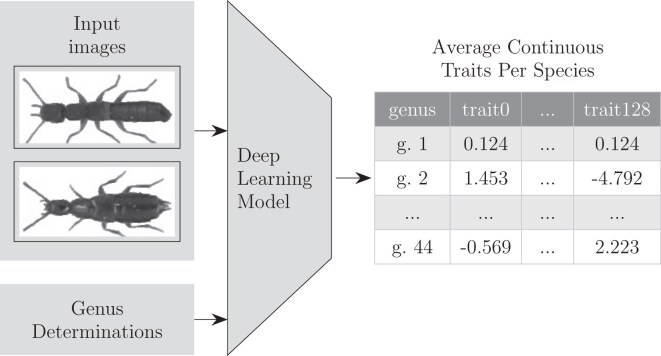
Pipeline for generation of continuous morphological traits per genus. A deep metric learning model was trained on dataset images. It used the genus determinations to pull specimens from the same genus closer together in space. A vector of 128 traits was generated from the model for each image. The average vector for each genus was then calculated.

**Fig 2:**
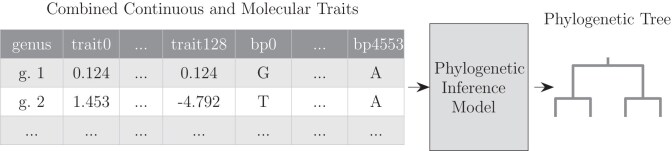
Pipeline of phylogenetic tree generation. First the continuous traits given by the deep learning model were combined with the molecular data. Both of these were then fed into the phylogenetic inference model which generates a tree.

The analysis pipeline was split into several steps (Figs 1 and 2). Each step is explained in the following sections.

#### Datasets for Phylophenomics

Deep learning methods require training datasets in order to learn representations of the data. Few datasets for phylogenetic inference from images exist at present. iNaturalist (Van Horn et al.^2021^), the butterfly dataset (Cuthill et al.^2019^) and Rove-Tree-11 ([Bibr CIT0032][Bibr CIT0032]) are the most notable of these. iNaturalist has the disadvantage that the images are in-the-wild (images taken from non-standardized angles, lighting, backgrounds, etc.) making morphological trait extraction more difficult and both iNaturalist and the butterfly dataset have the difficulty that the phylogenies derived from such datasets are not particularly fine-grained. Other datasets exist, however, are typically in-the-wild, making morphological analysis difficult, and/or only provide a shallow reference phylogeny, e.g. ([Bibr CIT0018][Bibr CIT0018]; Wu et al.^2019^; Goëau et al.^2021^; Gharaee et al.^2023^). Therefore, here we focus our attention on the Rove-Tree-11 dataset that provides over 13,000 dorsal images of rove beetles and associated 11 level deep reference phylogeny. Additionally, none of these datasets come with associated DNA sequences excluding BIOSCAN Gharaee et al. (2023), which has a particularly shallow taxonomy and varying insect orientations. In gathering the DNA data for the Rove-Tree-11 dataset we hope to open new avenues for phylogenetic research.

### Dataset

Images and the reference phylogeny used in this analysis were from Rove-Tree-11 ([Bibr CIT0032][Bibr CIT0032]). Rove-Tree-11 is a dataset of 13,887 segmented dorsal images of pinned beetles from the family Staphylinidae (rove beetles) housed in the Entomology collection at the Natural History Museum of Denmark, which includes species labels and an associated reference phylogeny based on the state-of-the-art knowledge in the field. The reference phylogeny was generated by expert coleopterists at the Natural History Museum of Denmark by combining recently published phylogenies from (Chani-Posse et al.^2018^; [Bibr CIT0005][Bibr CIT0005]; [Bibr CIT0091][Bibr CIT0091]; Żyła et al.^2022^).

**Fig 3:**
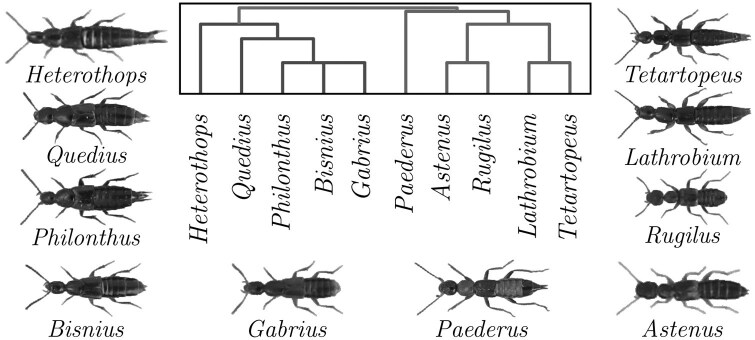
Subset of the reference phylogeny from the Rove-Tree-11 dataset, for the 10 genera with the most images in the dataset. Each leaf represents a genus. Example specimens from each of the genera are shown in black and white for reference. Reproduced from Hunt and Pedersen (2022) with permission.

**Fig 4:**
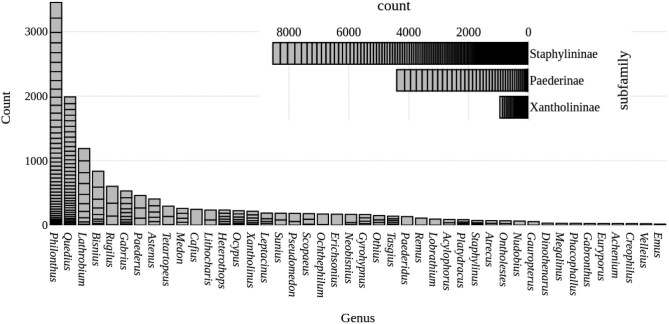
Distribution of specimens per genus (bottom left) and per subfamily (top right). Each slice in the stacked bar chart represents a different species within that genus. Reproduced from Hunt and Pedersen (2022) with permission.

The dataset was specifically released to explore deep-learning image based phylogenetic research, and as far as we know, it is the highest-depth publicly available dataset, which is why we focus our analysis on this dataset. Images in the dataset are highly standardized with background removed (Fig. 3). The distribution of the dataset on genus and sub-family level is skewed due to sample availability (Fig. 4).

### Molecular Data

The initial Rove-Tree-11 dataset does not include molecular data, and indeed these would be difficult if not impossible to obtain for the exact specimens included in the dataset as they are pinned specimens, likely to have degraded DNA ([Bibr CIT0054][Bibr CIT0054]). Therefore, molecular data were gathered from GenBank to augment the Rove-Tree-11 images and phylogeny. Since molecular data were not available for all individual species, we focused on covering examples from every genus. Genus *Velleius* from the original Rove-Tree-11 was downgraded to a subgenus of *Quedius* and therefore it was not present in our analyses. Seven genes were used in order to cover the majority of the genera present in the dataset: carbamoyl- phosphate synthetase (cadA and cadC), topoisomerase I (topo), arginine kinase (argK), wingless (Wg), mitochondrial protein-encoding COI (COI), and nuclear ribosomal 28S (28S). These were chosen based on their wide use and availability in molecular phylogenies focused on Staphylininae, Paederinae, and Xantholininae (Chatzimanolis et al.^2010^; Brunke et al.^2016^; [Bibr CIT0070][Bibr CIT0070]; Chani-Posse et al.^2018^; [Bibr CIT0091][Bibr CIT0091]; Jenkins Shaw et al.^2020^). All molecular data used in this analysis are provided in the Supplemental material. For protein-coding genes, the reading frame was found with Aliview ([Bibr CIT0047][Bibr CIT0047]). Individual gene fragments were aligned using MAFFT 7 ([Bibr CIT0039][Bibr CIT0039]). Trimming was performed on alignments using TrimAl 1.3 (Capella-Gutiérrez et al.^2009^) with -gappyout setting. The alignments were concatenated with FASconCAT-G ([Bibr CIT0044][Bibr CIT0044]). The best partition scheme and model selection were obtained under the Bayesian inference criterion using PartitionFinder 2.1.1 (Lanfear et al.^2016^). The following parameters were considered: "all models," the lengths of the branches were established so that they were "unlinked" and the search was established in the algorithm "greedy" (Lanfear et al.^2012^).

### Deep Learning Model

Following the methodology used by Hunt and Pedersen (2022) and Roth et al. (2020), we used a ResNet50 architecture (He et al.^2016^) pretrained on the ImageNet dataset (Russakovsky et al.^2015^) with 128 latent features and compared across various deep metric learning loss functions, described below. Models were trained for 50 epochs with the best checkpoint based on the validation results used to generate the continuous traits. For all training sessions, we fixed mini-batch size to 8 samples with gradient accumulation after 14 batches (an effective batch size of 112), a learning rate of 1e-5 with a step scheduler and a weight decay of 1e-4. Data augmentation was applied to improve the generalizability of the model. Details of the augmentations is available in the codebase on github. Unlike Hunt and Pedersen (2022) we trained the deep learning model on genus-level, since molecular data were not available at species level, and therefore, the total evidence analysis was performed at genus-level.

### Loss Functions

Various deep metric learning loss functions were compared, based on those used by Hunt and Pedersen (2022) and Roth et al. (2020). We focused on 6 popular deep metric learning loss functions out of thousands available. These include 4 ranking-based losses: triplet (Wu et al.^2017^), margin (Wu et al.^2017^), contrastive (Hadsell et al.^2006^), and multisimilarity (Wang et al.^2019^). Lifted was not used contrary to Hunt and Pedersen (2022) because it did not appear to properly converge. Furthermore, we also explored one proxy-based loss: proxyNCA (Movshovitz-Attias et al.^2017^) and one classification-based loss: arcface (Deng et al.^2018^). We adopted a beta parameter of 0.6 for the margin loss, consistent with prior work by Hunt and Pedersen (2022). Distance-based batch mining was applied to margin, contrastive, and triplet losses. Additional details can be found in Roth et al. (2020).

### Dataset Split

In order to train and evaluate the performance of deep learning methods a dataset is usually split into training, validation and test datasets. How the chosen dataset is split into these subsets can greatly affect the results obtained by deep learning algorithms ([Bibr CIT0077][Bibr CIT0077]). The original Rove-Tree-11 dataset was divided into training, validation, and test sets based on sub-family classification: Staphylininae for training, Paederinae for validation, and Xantholininae for testing, termed the "Clade" split. This arrangement aimed to assess a deep learning model’s capacity to learn phylogenetic relationships. However, we were interested in investigating whether utilizing a typical stratified classification split could enhance the model’s ability to discern phylogenetically significant features by exposing it to examples from all taxa during training. Hence, we introduced an additional dataset split called ’Stratified’. A stratified dataset split involves dividing the dataset into training, validation, and test sets in such a way that each set contains approximately the same percentage of samples of each target class as the original dataset, ensuring representative distribution of classes across each split.

For the stratified dataset split, we maintained the training, validation, and test set divisions but randomly allocated 70% of the images from each species to the training set, with the remaining 30% split equally between validation and test sets. This split conformed to standard practice in deep learning classification tasks. The new split’s details are provided in a Supplemental csv file for reproducibility purposes. No other model parameters were altered for this investigation beyond the adjustment in the training split.

### Maximum Parsimony Analysis

Due to software limitations, as mentioned earlier, we exclusively employed maximum parsimony for our analyses. This decision stems from the constraint that RevBayes is the sole Bayesian program supporting continuous traits; unfortunately, combining it with missing molecular data yielded convergence issues, as noted in an open issue on the RevBayes GitHub repository ([Bibr CIT0088][Bibr CIT0088]). We utilized TNT (Goloboff et al.^2008^) for maximum parsimony analyses, employing random addition sequences with TBR branch swapping across 100 replicates, followed by generating a Nelsen strict consensus and conducting bootstrapping with 100 repetitions. In total evidence analyses with maximum parsimony, we enforced monophyly within genera to enable tree score calculation at the genus level. Example scripts are included in the Supplemental material.

### Quantitatively Comparing Trees

To assess the quality of trees obtained through various methods, we employed quantitative techniques for comparing them to a reference phylogenetic tree. A common metric in phylogenetics is the normalized Robinson-Foulds (nRF) score ([Bibr CIT0066][Bibr CIT0066]). While advantageous for comparing trees of different sizes and depths, it has limitations; it relies on binary matching of tree edges, potentially inflating scores for trees with few errors. Alternatively, the align score (Nye et al.^2006^) computes the intersection over union for matched edges, providing a fairer representation of tree differences. However, it lacks normalization, making direct comparisons across trees of different sizes challenging. To address this, we normalized the align score based on the upper bound of matched edges. Both metrics are utilized: the align score is adept at detecting subtle differences between trees, while the nRF score is widely accepted and easier to interpret. Since our reference tree lacked branch length information, other methods focusing on branch length differences were irrelevant. Further details and comparisons between the two metrics are provided in Kuhner and Yamato (2014).

### Quantifying Phylogenetic Signal in Traits

Numerous methods are available to quantify the phylogenetic signal in traits, with a comprehensive comparison provided in Münkemüller et al. (2012). Among these methods, Abouheif’s Cmean ([Bibr CIT0001][Bibr CIT0001]) stood out as it does not rely on branch lengths and our phylogeny does not have branch lengths. This method evaluates the autocorrelation of trait values across the tree’s leaves and tests its significance against a randomly permuted dataset. Specifically, the function computes the sum of squared differences between adjacent trait values along the ordered list of leaves, divided by the total difference. The equation for Abouheif’s Cmean is shown in (1), where $y_{i}$ represents the trait value for species *i*, $y_{i+1}$ denotes the trait value for the neighboring species in the ordered list of leaves, and *N* indicates the total number of species. As a result, the function is normalized, and higher values indicate a stronger phylogenetic signal. The Cmean is defined as


$$Cmean(y)=1-\frac{\overset{N-1}{\underset{i=0}{\sum }}{\left(y_{i+1}-y_{i}\right)}^{2}}{2\overset{N}{\underset{i=0}{\sum }}y_{i}^{2}}\;.$$ (1)

### Gene Ablations

To assess the impact of adding genetic data on continuous traits results, we conducted maximum parsimony analyses using all possible combinations of the seven genes (argK, cadA, cadC, COI, 28S, topo, Wg) employed in this study. This entails 127 total combinations. Due to computational constraints, we focused on a single model with the lowest normalized Align Score — Triplet loss trained on the Stratified dataset split with a random seed of 4 to investigate how altering the number of genes affects total evidence results.

Given that not all genes were available for all genera, the resulting tree sizes varied in the molecular-only ablation study. As the normalized Align Score remains somewhat sensitive to tree size despite normalization, total evidence trees with more genera may have faced a slight disadvantage. To mitigate this, when calculating the normalized Align Score for total evidence trees compared to the reference tree, we excluded any genera lacking molecular data.

## Results

### Deep Learning Derived Morphological Traits: Phylogenetic Signal and theEffect of the Dataset Split

**Fig 5:**
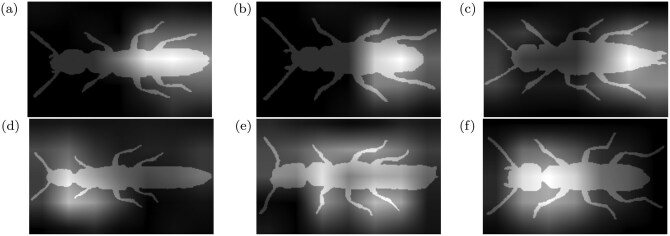
Examples of gradcam saliency maps (trait 30 from the stratified dataset with triplet loss, seed 4). Saliency maps are shown superimposed on the mask of the beetle with brighter pixel values indicating higher influence on the latent variable. Saliencies in the top row are from genera (a) *Atrecus*, (b) *Lithocharis* and (c) *Ontholestes*, these show the model focusing on the abdomen for this trait. And (d) *Gyrohypnus*, (e) *Nudobius* and (f) *Rugilus* demonstrate some counter examples for this trait. In the case of *Rugilus* (f) the model focuses instead on the neck region which is distinctively small in the *Rugilus* genus.

To demonstrate that the deep learning derived morphological traits have captured some phylogenetic signal, we calculated normalized Robinson Foulds (nRF) scores and normalized Align Scores (nAS) for trees inferred from the traits using maximum parsimony and varying dataset splits and loss functions (Table 1) and Cmean values for the traits of each model along with their p values (Table 2). Gradcam saliency maps applied to the segmentation masks show some focus on phylogenetically important areas (Fig. 5) (Selvaraju et al.^2017^).

**Table 1:** Tree Inference Results. Each row is the average of 5 runs. As a baseline for the scores, five randomly generated trees of this size gave a $nAS^{a}$ of 0.702±0.022 and a $nRF^{b}$ score of 0.993±0.007. Results are reported with 95% confidence intervals, using a student’s t-distribution. Best results in bold. Results within confidence interval of the best score are underlined.

		nAS			nRF		
Dataset Split	Loss Function		Average	Median		Average	Median
Clade	Arcface	0.596±0.020	0.627±0.022	0.625	0.947±0.019	0.975±0.017	0.969
Clade	Contrastive		0.629±0.017	0.637		0.967±0.005	0.969
Clade	Margin		0.615±0.045	0.616		0.975±0.017	0.970
Clade	Multisim.		0.506±0.034	0.512		0.877±0.043	0.897
Clade	Proxy		0.638±0.009	0.634		0.987±0.022	1.000
Clade	Triplet		0.560±0.045	0.563		0.901±0.065	0.903
Stratified	Arcface	0.595±0.031	0.692±0.042	0.708	0.913±0.028	0.992±0.022	1.000
Stratified	Contrastive		0.531±0.024	0.538		0.829±0.026	**0.815**
Stratified	Margin		0.596±0.036	0.597		0.928±0.045	0.925
Stratified	Multisim.		0.524±0.076	**0.505**		0.845±0.057	0.825
Stratified	Proxy		0.699±0.009	0.699		1.000±0.000	1.000
Stratified	Triplet		0.530±0.067	0.532		0.886±0.062	0.900

a normalized Align Score b normalized Robinson-Foulds

**Table 2:** Phylogenetic signal quantification using Abouheif’s Cmean. Each row is the average of the 128 traits of all 5 runs. Results reported with 95% confidence intervals, using a student’s t-distribution. Best results in bold. Results within confidence interval of the best score are underlined

						*P* value <0.05		
		Average Cmean		Maximum Cmean		with Cmean		
Dataset split	Loss function	Cmean	*P* value	Cmean	*P* value	>0.3	>0.5	>0.7
Clade	Arcface	0.107±0.010	0.219±0.019	0.480	0.001	9%	0%	0.0%
Clade	Contrastive	0.288±0.014	0.067±0.011	**0.794**	0.001	**46%**	**14%**	**0.6%**
Clade	Margin	0.188±0.012	0.118±0.015	0.610	0.001	21%	2%	0.0%
Clade	Multisim.	0.208±0.013	0.118±0.016	0.658	0.001	28%	4%	0.0%
Clade	Proxy	0.101±0.010	0.226±0.020	0.519	0.001	6%	0%	0.0%
Clade	Triplet	0.244±0.012	0.076±0.011	0.683	0.001	35%	5%	0.0%
Stratified	Arcface	−0.028±0.007	0.511±0.022	0.241	0.008	0%	0%	0.0%
Stratified	Contrastive	0.281±0.012	0.050±0.009	0.706	0.001	44%	8%	0.2%
Stratified	Margin	0.164±0.011	0.138±0.015	0.578	0.001	16%	1%	0.0%
Stratified	Multisim.	0.128±0.010	0.179±0.018	0.517	0.001	10%	0%	0.0%
Stratified	Proxy	−0.025±0.008	0.498±0.023	0.273	0.007	0%	0%	0.0%
Stratified	Triplet	0.232±0.011	0.076±0.012	0.645	0.001	31%	4%	0.0%

The positive examples (top) in Fig. 5 show that the deep learning derived traits can focus on phylogenetically important morphological features. In this case the model appeared to mainly look at the abdomen for this trait. However, the negative examples (bottom) show the model also focused on different areas in the same trait. *Rugilus* (f) shows the model focused on the narrow neck, a distinctive *Ruglilus* trait. For *Nudobius* (e) this trait looked at the majority of the beetle, showing that there is no direct relationship between the trait and the morphological area of interest, making the results difficult to interpret.

Dataset split appears to have no influence over the average results, and the best overall model on average according to the normalized Align Score used the cladistic dataset split (Table 1). We also see some preference for different loss functions. In particular contrastive, multisimilarity, and triplet losses provided the best results. The normalized Align and Robinson Foulds scores also showed a statistically significant improvement to randomly generated trees in 10 out of 12 models, further indicating that the continuous traits carried a phylogenetic signal, but also that model choice is important.

Abouheif’s Cmean values showed relatively low average trait values, and a high variation in the maximum phylogenetic signal per trait in the different models (Table 2). However, all maximum Cmeans had a significant *P* value (less than 0.05), indicating a strong and significant phylogenetic relationship obtained in some traits. The final three columns show the percent of traits which have a significant *P* value (less than 0.05) and a Cmean above a certain threshold, indicating the percentage of traits with a significantly strong phylogenetic signal. Of these, relatively few had a Cmean above 0.5. From this, the Cladistic dataset split appears to have a positive influence on the phylogenetic signal, with the cladistic split, contrastive loss model significantly outperforming the other models. It is interesting to contrast this with the results in Table 1 where the cladistic split, contrastive loss model performed worse than the average. This shows the strong influence of the metric on the results. When interpreting these results, we prefer to favor the align score as it directly measures the performance of phylogenetic inference from the traits, while Abouheif’s Cmean, as an autocorrelation function, is also influenced by the order the leaves are presented in the tree.

### Adding Molecular Sequences: Total Evidence Analysis

Here we report results from the total evidence analysis where we combined both deep learning derived morphological traits and molecular traits to complete the phylogenetic inference. We compared this combined result with using molecular data alone.

Unsurpisingly the dataset split does significantly affect the results, with the clade split providing the best Align Score on average (Table 3). This can be contrasted with the deep learning derived traits only results in Table 1, where the dataset split results were well within each other’s confidence intervals. Secondly, we can see that the results were on average slightly improved using total evidence compared with molecular traits only; however, 10 out of 12 models had average nAS scores within the confidence interval of the molecular only model, suggesting we should be cautious in drawing conclusions. That said, only two models had average nRF scores within the molecular only model’s confidence interval. This further highlights the discrepancies between these metrics, however, we believe the nAS is a more fair metric, for reasons explored in the methods section.

**Table 3:** Results of total evidence analysis – combining molecular data and deep learning derived morphological traits. Confidence intervals are 95% level based on 5 runs. As a baseline comparison for the scores, five randomly generated trees of this size would give a nAS of 0.702±0.022 and a nRF score of 0.993±0.007. Best results in bold. Results within confidence interval of the best score are underlined

			nAS			nRF		
Traits	Dataset split	Loss function		Average	Median		Average	Median
Molecular Only	-	-		0.141±0.017	0.147		0.382±0.040	0.405
Total Evidence	Clade	Arcface	0.128±0.006	0.139±0.021	0.138	0.320±0.010	0.337±0.024	0.333
Total Evidence	Clade	Contrastive		0.119±0.014	0.119		0.311±0.033	0.315
Total Evidence	Clade	Margin		0.127±0.017	0.127		0.319±0.025	0.315
Total Evidence	Clade	Multisim		0.135±0.024	0.136		0.316±0.045	**0.306**
Total Evidence	Clade	Proxy		0.127±0.013	0.126		0.324±0.041	0.315
Total Evidence	Clade	Triplet		0.121±0.023	**0.118**		0.309±0.031	0.324
Total Evidence	Stratified	Arcface	0.147±0.011	0.166±0.037	0.160	0.339±0.012	0.383±0.028	0.389
Total Evidence	Stratified	Contrastive		0.141±0.032	0.139		0.313±0.019	**0.306**
Total Evidence	Stratified	Margin		0.162±0.058	0.143		0.324±0.011	0.324
Total Evidence	Stratified	Multisim		0.135±0.016	0.133		0.337±0.026	0.333
Total Evidence	Stratified	Proxy		0.155±0.028	0.149		0.365±0.021	0.361
Total Evidence	Stratified	Triplet		0.121±0.012	0.119		0.310±0.017	0.315

### Qualitative Tree Comparison

**Fig 6:**
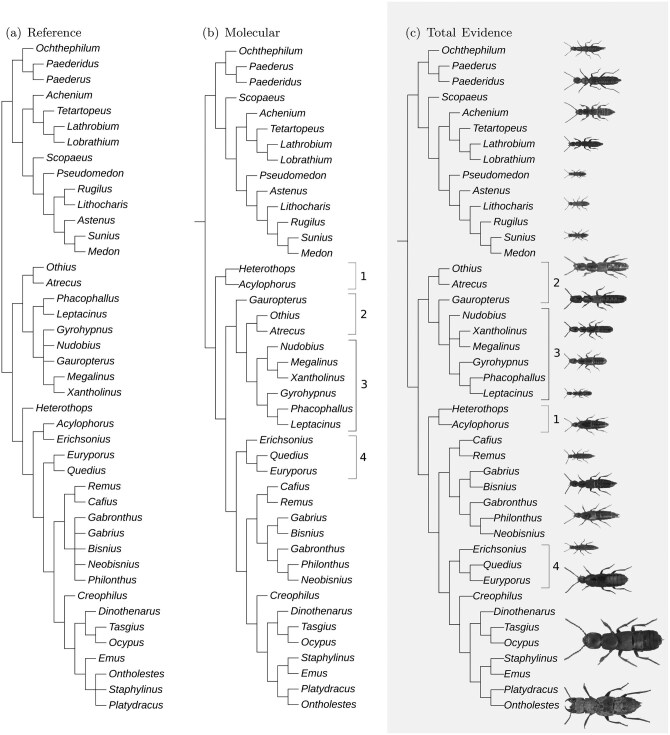
Comparison of a) reference phylogeny, b) best molecular-only tree and c) best total evidence tree. Differences between best molecular-only and best total evidence tree highlighted by indicating the controversial groups 1,2,3,4 on both trees. Plots produced in part using iTOL (Letunic and Bork, 2021)

Here we evaluated the differences between the gold standard tree, best molecular tree, and best total-evidence tree (Fig. 6). Differences between them are labelled as groups 1–4 and examined as follows:

The total evidence tree (Fig. 6c) correctly placed *Heterothops* and *Aclyophorus* in Staphylininae. while the molecular only tree (Fig. 6b) claded them as a sister group to Xantholininae and Staphylininae.
The total evidence tree (Fig. 6c) placed *Gauropterus* inside the majority of Xantholininae, as expected, while the molecular only tree (Fig. 6b) erroneously placed *Gauropterus* outside the majority of Xantholininae.
The total evidence tree (Fig. 6c) pushed these clades to be unresolved, closely reflecting our current empirical data about these clades while the molecular-only tree (Fig. 6b) undesirably resolved these clades
The total evidence tree (Fig. 6c) erroneously nested *Quedius* and *Euryporus* inside the tribe Staphylinini, while the molecular only tree (Fig. 6b) correctly placed them outside Staphylinini.

In total, 3 out of 4 controversial groups in this example are better placed by the total evidence tree.

### Varying Amounts of Molecular Sequences: Gene Ablation Study

**Fig 7:**
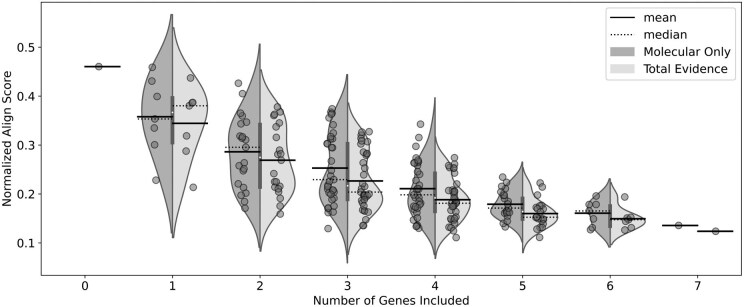
Combined Violin plot/Strip chart of the effect of including different gene combinations on the normalised Align Score. In each column the results to the left (darker) are for molecular-only ablations, and to the right (lighter) are for total evidence ablations. Each point represents an individual result.

Changing the number of genes included in the analysis affects the results (Fig. 7). We notice that the total evidence ablation results are slightly improved on average compared to the molecular-only results. However, there is a large variation in the normalized Align Score. This variation is decreased as we add more genes, which also makes sense as the number of possible combinations decreases and the algorithm has more data to converge on.

We see disagreement between the nRF and nAS scores, and in this case we follow the nAS scores as they are more stable (Table 4). The nAS indicates that the best model includes continuous deep learning derived traits and the 4 genes 28S, CADb, COI, and Wg. On average we can see both from Table 4 and from Fig. 7 that the nAS in the total evidence results were generally lower and therefore better than they were in the molecular only results, further suggesting that deep learning derived continuous traits can add to phylogenetic analyses.

**Table 4:** Best subset of genes^c^ given the number of genes. Best results in bold

	Molecular only			Total evidence		
No. genes	Best gene combination	nAS	nRF	Best gene combination	nAS	nRF
0	-	-	-	-	0.461	0.846
1	28S	0.228	0.433	28S	0.214	0.420
2	28S, ArgK	0.171	0.362	28S, ArgK	0.159	0.408
3	28S, COI, Wg	0.129	**0.275**	28S, ArgK, topo	0.135	0.286
4	28S, COI, topo, Wg	0.130	0.314	28S, cadB, COI, Wg	**0.111**	0.278
5	28S, ArgK, cadB, COI, Wg	0.133	0.432	28S, cadB, COI, topo, Wg	**0.111**	0.288
6	28S, ArgK, cadA, cadB, COI, topo	0.127	0.342	28S, ArgK, cadB, COI, topo, Wg	0.126	0.324
7	All	0.136	0.378	All	0.124	0.315

Genes used in this analysis: nuclear ribosomal 28S (28S), arginine kinase (ArgK), carbamoyl- phosphate synthetase (cadA and cadC), mitochondrial protein-encoding COI (COI) topoisomerase I (topo) and wingless (Wg)

## Discussion

### Deep Learning Derived Morphological Traits: Phylogenetic Signal

All of the models on average had a better Align Score than random trees, and 10 out of 12 models were outside of the confidence intervals for the random trees, indicating that the deep learning derived traits can indeed carry a statistically significant phylogenetic signal (Table 1). However, that signal is still difficult to directly interpret as demonstrated by the saliency maps in Fig. 5. While applying saliency maps that are normally used in classification to latent variables is itself questionable, we can see that this deep learning derived trait does appear to primarily focus on a single body part; however, the saliency map can deviate from this, and while we can explain some of this through our knowledge of the distinctive traits (like the slim neck of *Rugilus*), others are difficult to interpret. We assume that this, in part, demonstrates the dependencies between these traits, and potentially demonstrates that further exploration is necessary into explaining these traits. We put no constraints on the traits to be independent, and indeed we can see that many traits focused on the same area for example from the same genera. It could be that interpreting such models could be made easier by the use of disentangled networks. We discuss this further in the future work section. Initial explorations of simply applying PCA to the deep learning derived traits did not improve the cmean results (see Supplementary material), suggesting that if the traits are entangled, they probably follow a non-linear entanglement and therefore require non-linear manifold learning methods (Mysling et al.^2011^).

### The Effect of the Dataset Split

Our results indicate a preference for the cladistic split (Tables 1 and 3), but in Table 3 this preference is significant. This is interesting as we expected to see the opposite – that the difference would be more pronounced when the molecular traits have no influence on the results. We are also surprised that the preference is not for the stratified split. We expected that allowing the model to learn from all subfamilies would improve the model’s ability to learn phylogenetically important traits for other groups. However, these results are encouraging, in the sense that they indicate that models trained on one clade may be highly successful in inferring relationships in unseen clades, reducing the necessity for retraining the model. However, further investigation into this phenomena on a variety of datasets would be required to firm up a conclusion on this aspect.

### Loss Functions

When comparing across loss functions in Tables 1 and 3 we see a clear preference for contrastive, multisimilarity and triplet losses. This is in line with results obtained in Hunt and Pedersen (2022). What is interesting is that the results from Abouheif’s Cmean and the total evidence analysis in Tables 2 and 3 suggest a strong preference for the contrastive loss, which is not necessarily reflected in the results in Table 1 where we infer the phylogeny directly from the morphological traits. A high value of Abouheif’s Cmean is highly correlated to deeper phylogenetic relationships. Therefore one hypothesis could be that the contrastive loss is better at picking up deeper (tribe-level and above) phylogenetic relationships, but not so good at picking up more shallow relationships, making it result in traits which have a relatively high Cmean, but relatively poor overall tree. However, since molecular data is quite good for picking up shallow relationships, when combined with these morphological traits, this could result in a good total evidence tree. This is one potential explanation, but this would need to be investigated further and is outside the scope of this analysis.

### Qualitative Tree Comparison

The total-evidence best tree topology (Fig. 6c) reveals improved placements of the *Heterothops* and *Gauropterus* inside Staphylininae, as well as it reflects the uncertainty in the unresolved position of the *Xantholinus* and *Megalinus* clades. However, it places the *Erichsonius*+(*Quedius*+*Europorus*) clade inside the tribe Staphylinini. Alternative phylogenetic placement of these groups suggests that deep learning derived traits improved phylogenetic resolution at deeper nodes, in our case at the subfamily level, but failed with more terminal resolution, in our case at the tribal level.

### Gene Ablation Study

The total-evidence analysis with four genes (28S, CADb, COI and Wg) provides the best result of the ablation studies (Fig. 7 and Table 4). There is significant variation in the results when a single gene is added, reinforcing the notion that gene choice is important for phylogenetic analyses (Fig. 7). We also see that good results can be obtained with relatively few genes, and that this result is not drastically affected by adding deep learning derived morphological traits (Fig. 7). We can further say that deep learning derived morphological traits can indeed improve the phylogenetic analysis, with mean values below that of the molecular results, however this result should be used with caution as for the gene ablation study we used traits from the model which performed the best. Therefore a comparison of different models on the data of interest should be completed to make further conclusions from these analyses.

## Conclusion

Quantitative morphological characteristics, extracted through the application of deep learning techniques applied to images of pinned insect specimens produced for mass collections digitization purposes, have been demonstrated to possess phylogenetic relevance. These traits, when integrated into molecular phylogenies, have the potential to augment the phylogenetic framework in a comprehensive total-evidence based approach, offering the possibility of incorporating species lacking molecular data into phylogenetic trees. However, the improvement of phylogenetic reconstructions by the inclusion of such morphological data derived through deep learning methodologies remains minimal. While this approach has shown promise, scaling up its implementation is still not feasible for 2 reasons. First, the phylogenetic signal of the deep learning derived traits, at least in our dataset, was not strong enough to justify the additional effort in gathering the data. Second, the effort required for our image-based model testing, even though likely lesser than an effort by the expert to assemble a traditional morphological phylogenetic matrix, is still significant. Despite these challenges, as will be elaborated in the section on future research directions, we stress the opportunities for removal of both impediments. It is conceivable to amplify the phylogenetic signal automatically extracted from the collections specimen images via improved models, targeted loss functions or transfer learning. At the same time improved imaging, image processing and on-going optimization of these steps should enhance the data acquisition and thus large-scale non-destructive use of the digitized collections.

## Future Work

Several promising directions exist within the developing domain of deep metric learning, which could improve the phylogenetic signal obtained by these models. Firstly, with natural history museums worldwide embarking on the digitization of their collections ([Bibr CIT0012][Bibr CIT0012]; Hedrick et al.^2020^; Popov et al.^2021^; [Bibr CIT0002][Bibr CIT0002]; Johnson et al.^2023^), an expansion of publicly accessible data to nearly complete sampling of big taxa is anticipated. Given that the performance of deep learning models is generally enhanced by training on more representative datasets (Sun et al.^2017^), it is anticipated that the increased availability of image datasets in this domain will significantly enhance the efficacy of resultant models.

Secondly, the domain of deep learning is experiencing rapid evolution, characterized by the emergence of numerous sub-fields. It is still unclear which improvements to deep learning models may significantly increase the extracted phylogenetic signal. Two areas, in particular, hold significant promise. A growing body of research (Eddahmani et al.^2023^) is investigating the enforcement of independence among continuous traits, which could lead to more explainable traits and enable their statistical dissociation. Another area of interest pertains to the application of variational autoencoders to encourage the continuous trait space to conform to specific distributions (e.g., a normal distribution), which has been demonstrated to improve clustering outcomes in various instances ([Bibr CIT0042][Bibr CIT0042]). Invoking the central limit theorem, the normal distribution possesses intrinsic appeal in biological contexts and may facilitate a better structured trait space. More work also should be done into how to measure the phylogenetic signal in these models.

Thirdly, the integration of deep learning-derived molecular embeddings with morphological embeddings in phylogenetics remains an underexplored avenue, despite significant investment in the development of deep learning approaches for molecular traits (Mo et al.^2024^).

Fourthly, there is a necessity for further investigation into the quantification and explanation of deep learning-derived traits. The identification of loss functions capable of incentivizing models to discern phylogenetically pertinent traits remains elusive. Moreover, existing metrics for quantifying the informational content of traits, such as Abouheif’s Cmean or align scores, lack intuitiveness. While saliency maps offer a generalized explanation of model behaviors, their applicability to latent variable models is not straightforward, and a comparison of different latent variable saliency methods should be completed.

Lastly, we advocate for the conceptualization of deep learning-derived traits as distributions rather than singular values. Morphological traits are traditionally represented as binary values across clades; however, we contend that recognizing each species as a continuous distribution of traits could hold substantial value. The employment of continuous variables in conjunction with Bayesian methodologies appears particularly conducive to this perspective.

## Environmental Footprint

Using carbontracker (Anthony et al.^2020^) we estimate that the training of each deep learning model in this paper used 1.32 kWh of power (based on one full model run), translating to 192 g of $CO_{2}$. The full published deep learning results from this paper therefore produced an estimated 11.5 kg of $CO_{2}$. The carbon production from the phylogenetic models and from early experimentation is not included in this estimate.

## Acknowledgments

Many people have contributed to this work, but in particular, we would like to thank François Lauze for his advice throughout this process and David Gutschenreiter, Søren Bech and André Fastrup for their work gathering images and completing segmentations on the Rove-Tree-11 dataset. We would like to express our gratitude to ChatGPT for its assistance in refining the text.

## Supplementary Material

Data available from the Dryad Digital Repository: http://dx.doi.org/10.5061/dryad.9cnp5hqqq.

## Funding

This work is partially supported by the University of Copenhagen Data+ funding (Strategic funds 2023) under the project "Expanding the Tree Life through a digital view of museum collections (PHYLORAMA)" and a Villum Experiment Grant under the project "Baltic Amber Enigma."

## Data Availability

The data underlying this article are available at http://doi.org/10.17894/ucph.39619bba-4569-4415-9f25-d6a0ff64f0e3 for the Rove-Tree-11 dataset and in the article’s dryad repository (doi: 10.5061/dryad.9cnp5hqqq) for the further molecular data and associated genbank accession numbers, example inference code, all generated trees, and stratified dataset split. All trained model runs and extracted trait matrices are available in the following erda repository https://erda.ku.dk/archives/440063cabdb1789ad82f31366c926b4e/published-archive.html. The reference tree, best molecular tree and best total-evidence tree can be found on TreeBASE at http://purl.org/phylo/treebase/phylows/study/TB2:S31300?x-access-code=397cc12bd8047bf52b312b4743f23e2b&format=html. The code used in this analysis is available on github https://github.com/robertahunt/Revisiting_Deep_Metric_Learning_PyTorch, commit a6654453c3b7785a17511255e02c468c53fe6f5d, forked from Roth et al. (2020).
